# Lady Visitors to Her Majesty's Prisons

**Published:** 1898-09-24

**Authors:** 


### Modern Sociology.

### LADY VISITORS TO HER MAJESTY'S PRISONS.

The *Daily Chronicle* for September 9th contains a statement by the Prison Commissioners, which is of much greater importance than might at first sight be inferred. They intimate that "they are concerned in the welfare of female prisoners, and are anxious to secure the services of discreet and judicious ladies to visit them at regular periods." In these columns, while dealing with prison discipline generally, we have repeatedly alluded incidentally to the valuable results which would accrue from the appointment of rightly qualified lady visitors to every prison in the country; but it is only now that the subject can be usefully treated in detail, when the agitation for prison reform has decided the authorities on the employment of visitors, not only in isolated cases as hitherto, but as a definite part of the whole system of prison management. It is, perhaps, scarcely necessary to point out the invaluable benefits which are certain to result from the universal adoption of the plan. What has already been done in the few instances where lady visitors have been allowed to work may sufficiently prove this. We might instance the female convict prison at Aylesbury, where Adeline Duchess of Bedford and her friend Lady Battersea are working in the most admirable manner, with great success, among the women undergoing long terms of imprisonment for serious crimes. We have it also on the testimony of one of the earliest workers, who has been attached to a prison for many years, that it is rare, indeed, to meet with a single case which cannot be, to some extent at least, benefited by the visitor's influence.

We must spare a few lines of our space to give a typical instance which has occurred very recently. A young woman who had gone through long periods of incarceration, where her violence and insubordination had made the lives of the officers a burden to them, was sent to a prison where there was a lady visitor. When first left alone with her the girl, only eighteen years of age, was fierce and sullen to the last degree, but gradually softened, and told how she had run away from a godless home at the age of thirteen, and been on London streets ever since. She "had not a friend in the world," she said. That first interview was devoted to proving that she had at last found a friend in the visitor. On the next occasion when they were to meet she was in punishment in her cell for having given way to a paroxysm of fury, in which she had tried to fight another prisoner in the exercise ground, had struck the matron, and, to use the words of the relater of her doings, "had squared up at the chief warder himself." Nevertheless, after half an hour spent with her new friend, the poor, wild girl pathetically exclaimed that she had had "such a bad bringing up," and, flinging out her hands, she said she would do whatever the lady wished; she might dispose of her in any way she pleased. As a result, on her discharge from prison she was placed in a Home, from whence she wrote to her friend begging her to forgive her former bad behaviour, and saying she would try to reward her for her kindness by her future good conduct. The utility of the system being no longer in question, we proceed to deal with the methods by which it should be carried out. The qualifications most necessary for a prison visitor are first, absolute loyalty to the authorities and honourable obedience to the minutest rule of the recognised code, an unswerving adherence to a high standard of principle, never palliating crime, and a deep though not sentimental sympathy with the individual prisoners. The Prison Commissioners, in the notice we have quoted, suggest lectures being given by the visitors to select classes. We do not wish to deny the advantage there may be in such methods of instruction, but it is a plan which, according to the most experienced matrons, has serious drawbacks. There is a certain *ésprit de corps* amongst these lawless women, who have been accustomed to mock at religious topics and to hold unconsciously most atheistical beliefs, and when they are assembled to listen to what they term the "lady's preachment" they like to show one another their absolute indifference to it, and to jeer and scoff at all that is said by means of signs and whispers. We hold most strongly to the conviction that there is but one way in which visitors can obtain a true and salutary influence over criminal women, and that is by seeing each one entirely alone, and by showing a sympathetic interest in her personal history. When this is manifested with simple, friendly words, and in a kindly manner, it is astonishing how quickly the poor outcast is led to find comfort in pouring out the details of all her trials and sorrows and delinquencies; and when a tolerably clear diagnosis of her mental state and her case generally is thus obtained, it becomes comparatively easy to devise means for her reform and future well being. Of course, the visitor must be well on her guard against hypocrisy and deceit and an inveterate habit of lying. She will be wise if she regards the first statements made to her as a tissue of falsehoods, but she has the police reports to give her an accurate account of all criminal facts in the prisoner's career, and by careful handling of the women's feelings, and a sifting of facts with judicial clearness which she has not mental capacity to resist, she is soon led to see it will be best for her own self-interest to tell the simple truth, and she generally does so. These interviews can always, of course, take place in the prisoner's cell, but there is considerable advantage in the lady visitor having a room in which the prisoners can be brought to her one by one. They feel themselves there, as it were, in a new sphere which invites free intercourse, and it is a much-coveted holiday for them to be received in it as her guests, and to be allowed in winter to sit near a fire, which at no other time they can ever see. The arrangements for lady visitors in large prisons, where several are appointed, require most serious amendment. In some of these each lady takes the duty for one week, or perhaps fortnight, at a time, and then gives way to her colleague. These ladies, being human, are all of different temperaments and views, and each one reverses the plans of her predecessors and starts on some new development. The result, of course, is that individual cases become involved in a hopeless imbroglio, in which no good can be accomplished. Where the prison is small enough to allow of it there ought to be only one visitor, who, with energy and great regularity of attendance, can effect much more than might be supposed possible; but when the number of prisoners is so great as to necessitate several workers they ought to be told off into separate companies, of which one ought to be assigned to each visitor as her sole charge, free from the interference of any of her colleagues.

